# Urban vs Suburban: Is the Door-to-Balloon Time Affected by Geographic, Socioeconomic, or Racial Differences? A Tale of Two Campuses

**DOI:** 10.1155/2020/8367123

**Published:** 2020-09-08

**Authors:** Marc Zughaib, Patrick Ters, Robby Singh, Marcel Zughaib

**Affiliations:** Providence Hospital, Michigan State University, Southfield, MI, USA

## Abstract

**Background:**

In 2004, the ACC/AHA released guidelines in the treatment of ST-segment elevation myocardial infarction (STEMI) within a time window from the time a patient physically enters the hospital to the time of percutaneous coronary intervention (PCI). This time window is defined as the door-to-balloon time (DTB) and is recommended to be under 90 minutes to improve patient mortality. To add another layer of complexity, patients with varying socioeconomic status and racial differences experience large disparities in health. Our institution provides care for patients in two locations separated by approximately 30 miles within the Detroit metropolitan area. We aimed this study to investigate any differences between DTB times of our two campuses (urban versus suburban population) as well as any differences in the components that comprise DTB times.

**Methods:**

We retrospectively collected data on all patients who presented to either Campus 1 or Campus 2 with a STEMI from 2016 to 17. DTB times, demographical, temporal, and anatomical data were collected and analyzed. Our search included 169 patients who met the full inclusion criteria.

**Results:**

The combined average of the overall DTB time for both campuses was 81 minutes, 15 seconds (95% CI: 78:05, 84:25). The average DTB time in Campus 1 was 78 minutes and 41 seconds (95% CI: 73:05, 84:18) versus 82 minutes and 46 seconds (95% CI: 78:55, 86:38) for Campus 2 (*p*=0.24). There were no statistically significant differences between either campuses within the separate metrics that comprise DTB times.

**Conclusions:**

Our study demonstrated that we have been able to provide high-quality care to all of our patients presenting with STEMI at either campus, regardless of socioeconomic differences in the populations they serve. Additionally, each campus has demonstrated DTB well below the nationally recommended guidelines.

## 1. Introduction

An acute ST-elevation myocardial infarction (STEMI) is an event in which transmural myocardial ischemia results in myocardial injury or necrosis [1]. The estimated annual incidence of myocardial infarction (MI) in the United States is 550,000 new cases per year and 200,000 recurrent cases per year [2]. Recently, it has been estimated that the incidence rates of STEMI decreased appreciably (121 to 77 per 100,000), whereas those of NSTEMI increased slightly (126 to 132 per 100,000) from 1997 to 2005 [3].

A patient presenting with STEMI represents an acute medical emergency requiring immediate complex care coordination to achieve narrow timeliness guidelines for myocardial reperfusion [4]. In patients presenting with STEMI, primary percutaneous coronary intervention (PCI) is the preferred strategy of reperfusion therapy. In 2004, the ACC/AHA released guidelines in the setting and treatment of STEMI. This guideline recommended a time window from the time a patient physically enters the hospital to the time of PCI to be under 90 minutes [5, 6]. This time window was subsequently termed the “door-to-balloon time” or “DTB.” Hospitals and medical institutions have implemented a variety of quality protocols within their own systems [7]. Ongoing efforts are recommended to improve the DTB times, which improves the patient-specific mortality overall. There have been several studies that demonstrated an inverse correlation between DTB time and risk of mortality [8–12]. Currently, it is estimated that almost 90% of patients presenting to a PCI-capable hospital without a clinical reason for delay have a DTB time ≤90 minutes [13, 14].

Our institution provides care for patients in two locations within the Detroit metropolitan area with primary PCI capabilities. These campuses are separated by approximately 30 miles and strive to deliver world-class excellence and quality care to patients, particularly those presenting with life-threatening events. Overall, both campuses report approximately 125 patients per year presenting with STEMIs. Campus 1 is an older institution but also has more beds and advanced services when compared with Campus 2. However, the main difference between our institution's campuses is in regards to the population they serve. Campus 1 serves patients in an urban population with a median income of about $54,000, while Campus 2 serves a more suburban/rural population with a median income of $92,000 [15]. Campus 1 is located in a city in which 70.3% of the residents are African American, while Campus 2 reported 8.5% of the residents as African American [15].

We aimed to perform a retrospective study investigating our own DTB times in an effort to improve our protocols and patient outcomes. We wanted to investigate any differences between the two campuses (urban versus suburban population) as well as any differences in the components that comprise DTB times. These components include Door to ECG, ECG to timing of the page to the interventional cardiologist, time of the page to time the patient was on the table in the cardiac catheterization lab, and time from the patient on the table to deployment of the balloon within the culprit lesion.

## 2. Methods

We retrospectively collected data on all patients who presented to either Campus 1 or Campus 2 with a STEMI from 2016 to 17. DTB times, demographical, temporal, and anatomical data were collected and analyzed. All patients with ST elevation from other causes were excluded. Patient charts were systematically read to determine the exact time of chest pain onset, presentation to ED, time of ECG, page to the interventional cardiologist, and DTB times.

Initially, our search included 220 patients between the two campuses, and 169 met the full inclusion criteria. Patients were excluded if the DTB could not be determined, if ECG or page to the cardiologist occurred prior to presentation in hospital, or if the patient was already admitted to the inpatient services of the hospital. Statistical analyses were performed with Microsoft Excel. Continuous data were analyzed using 2 tailed *t* tests, and categorical data were analyzed using chi-squared tests.

## 3. Results

Our study collected data about all STEMI cases occurring on both campuses for two calendar years (2016 through 2017). There were 169 patients presenting with a clinical scenario and ECG criteria consistent with STEMI that met our inclusion criteria. Campus 1 included 58 patients, and Campus 2 had 111 patients. The combined patient population was on average 63.76 years old at time of STEMI diagnosis (95% CI: 61.87, 65.64) (Table 1). Average BMI was 28.78 (95% CI: 27.98, 29.58). Campus 1 and Campus 2 did not have a statistical difference in the age of their patients at time of STEMI diagnosis (*p*=0.48), BMI (*p*=0.15), or distribution of gender (male *p*=0.63, female *p*=0.46) (Table 2). There were combined 120 males (70.01%) and 49 females (28.99%) within our studied population. Overall, there were 121 Caucasians (71.6%), 29 African Americans (17.16%), and 19 patients with other/unknown race (11.24%) included in our study. However, there were significant racial differences between the two locations as Campus 1 had a population that was 34.48% Caucasian, 62.07% African American, and 3.45% other/unknown. Campus 2 reported 94.59% Caucasian, 0.9% African American, and 4.5% other/unknown race (*p* < 0.001).

There were 40 (23.67%) patients diagnosed with coronary artery disease (CAD) prior to STEMI diagnosis. There were 96 (56.81%) patients diagnosed with hypertension (HTN), 41 (24.26%) with diabetes mellitus (DM), 7 (4.14%) with chronic kidney disease (CKD), 85 (50.3%) with a history of tobacco abuse, and 47 (27.81%) with a history of alcohol use. There were no statistical differences observed in these variables between the patients presenting at either campus.

**Figure 1:**
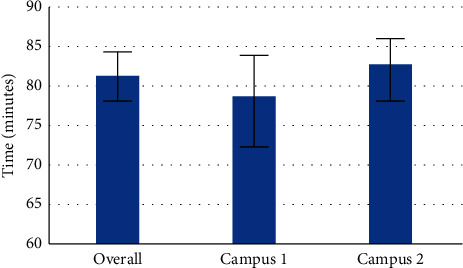
Comparison of door-to-balloon times between the overall population, Campus 1, and Campus 2. Error bars represent 95% confidence intervals.

The combined average of the overall DTB time for both campuses was 81 minutes, 15 seconds (95% CI: 78:05, 84:25) (Table 3, Figure 1). The average DTB time in Campus 1 was 78 minutes and 41 seconds (95% CI: 73:05, 84:18) versus 82 minutes and 46 seconds (95% CI: 78:55, 86:38) for Campus 2 (*p*=0.24) (Table 4). The shortest DTB was 43 minutes, while the longest DTB was 2 hours and 52 minutes (Table 5).

**Figure 2:**
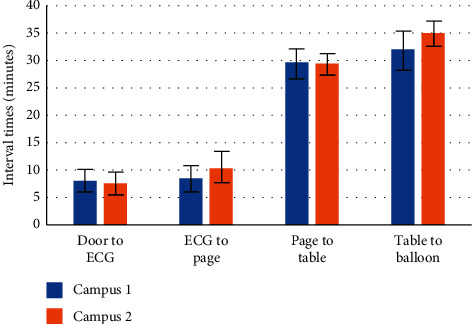
Comparison of door-to-balloon intervals between Campus 1 and Campus 2. Vertical axis represents minutes. Error bars represent 95% confidence intervals of the mean times.

The average time from a patient entering the emergency department to receiving an ECG across both campuses was 7 minutes and 50 seconds (95% CI: 6:29, 9:11) (Table 3). Campus 1 reported an average door-to-ECG time of 8 minutes and 11 seconds (95% CI: 6:20, 10:02), while Campus 2 had an average time of 7 minutes and 39 seconds (95% CI: 5:49, 9:29) (*p*=0.68) (Table 4, Figure 2). The shortest time within this door-to-ECG interval was 1 minute, while the longest instance was 1 hour and 9 minutes (Table 5).

The average time from a patient receiving an ECG to an outgoing page to an interventional cardiologist was 9 minutes and 51 seconds (95% CI: 7:55, 11:47) (Table 3). Campus 1 reported an average ECG to page time of 8 minutes and 32 seconds (95% CI: 6:29, 10:36), while Campus 2 had an average time of 10 minutes and 32 seconds (95% CI: 7:48, 13:16) (*p*=0.25) (Table 4). The shortest time in this interval was 1 minute, while the longest instance was 2 hours and 7 minutes (Table 5).

The average time from catheterization laboratory activation to patient arrival at catheterization laboratory table was 29 minutes and 25 seconds (95% CI: 27:42, 31:08) (Table 3). Campus 1 had an average time from catheterization laboratory activation to patient arrival at catheterization laboratory of 29 minutes and 46 seconds (95% CI: 26:43, 32:48), while Campus 2 had an average of 29 minutes and 26 seconds (95% CI: 27:19, 31:33) (*p*=0.86) (Table 4). The shortest time in this interval was 8 minutes, while the longest time was 1 hour and 9 minutes (Table 5).

The average time from the patient arrival at catheterization laboratory table to the time the balloon was deployed across the culprit lesion was 34 minutes and 9 seconds (95% CI: 32:19, 35:59) (Table 3). Campus 1 had an average time from the patient laying on the table in the catheterization lab to the time the balloon was deployed across the culprit lesion of 32 minutes and 12 seconds (95% CI: 28:54, 35:31), while Campus 2 had an average of 35 minutes and 10 seconds (95% CI: 32:59, 37:20) (*p*=0.15) (Table 4). The shortest time during this interval was 14 minutes, while the longest was 1 hour and 40 minutes (Table 5).

## 4. Discussion

Door-to-balloon (DTB) time is a complex outcome that is potentially affected by many different prognostic factors and care processes. Since the release of the updated ACC/AHA guidelines in the 2004, hospitals and ED units have updated internal protocols and quality improvement projects to reduce the DTB times to the meet appropriate timing of less than 90 minutes. Multiple strategies have been developed, including increasing public awareness of STEMI signs and symptoms, encouraging calling EMS, performing prehospital en-route ECGs, transmitting ECG tracings to emergency medicine physicians, allowing emergency medicine physicians to activate the catheterization lab, and increasing general awareness of streamlined protocols.

The strongest evidence, supported by the largest number of credible studies, is for activation of the catheterization laboratory using emergency medicine physicians rather than cardiologists, greater use of prehospital ECGs, and use of data monitoring/feedback. The same meta-analysis stated reasonable evidence also exists for establishing a single-call system for activating the catheterization laboratory, setting the expectation that the cardiac catheterization team will be available within 30 minutes after being paged, and having an organizational environment with strong senior management support and team-based culture to foster and sustain organizational changes directed at improving door-to-balloon time [16]. As part of our institution's long-standing protocols, the monthly review of all STEMI cases has been implemented. Thorough review of all STEMI cases that did not meet the 90 minute DTB time recommendation initiated a root cause analysis (RCA). This RCA breaks down the DTB time into individual components such as door to ECG, ECG to page to physician, time from page to transfer to the cardiac catheterization table, and time from placement on cardiac catheterization table to angioplasty. This multidisciplinary process includes EMS teams, ED physicians, ED staff, catheterization lab staff, and interventional cardiologists and allows for the opportunity to continue identifying areas for potential quality improvement.

Although there are continuous improvements in medical care and disease prevention, health disparities persist and may be increasing for chronic conditions such as obesity, cardiovascular disease, and cancer [17, 18]. Economically disadvantaged racial and ethnic minorities and populations of all races with low socioeconomic status and significant racial disparities experience large disparities in health [19]. Our institution has one campus serving the majority of patients with a lower socioeconomic status compared with the suburban campus. Our study demonstrated we have been able to provide high-quality care to all of our patients presenting with STEMI at either campus, regardless of socioeconomic differences in the populations they serve. Many of these STEMI patients can also experience heart failure as a consequence, and it is always important to consider the psychosocial determinants of health when managing these patients [20].

Even though there was no statistically significant difference in results between the two campuses, we acknowledge a few limitations within our study. First, there is a possibility that our analysis may be underpowered as to the number of patients included. Consequently, there were trends noted in the analysis that were not statistically significant and a larger number of patients may have lead to statistically significant results. Interestingly, the two campuses are staffed by the same groups of emergency physicians and interventional cardiologists although there are different cardiac catheterization lab staff in the two hospitals. Despite this, we noted that the greatest difference between the two campuses, albeit not statistically significant, was in door-to-ECG time which was longer in campus 1, the urban institution. Hence, the intake process at this institution may benefit from further investigation and improvement in workflow to expedite the door-to-ECG time.

## 5. Conclusion

The nationally recommended door-to-balloon time <90 minutes remains an important metric that hospitals across the nation strive to achieve and improve upon. We evaluated two different hospitals and despite demographic, socioeconomic, geographical, medical, and hierarchical differences between the 2 hospital campuses and the populations they serve, we found no statistical difference between DTB times. Our institution demonstrated average DTB times for two campuses below the national recommendation of 90 minutes. However, there remain areas of improvement, as several of the most outstanding institutions are now regularly achieving DTB times under 60 minutes [21, 22]. We believe this should be our next goal as we move forward in improving our ability to provide the highest quality of care to all of our patients.

## Figures and Tables

**Table 1:** Baseline demographic characteristics for entire population (*n* = 169).

	Mean (*n*)
Age at STEMI (95% CI)	63.76 (61.87, 65.64)
BMI (95% CI)	28.78 (27.98, 29.58)

Gender	
Male (%)	120 (71.01)
Female (%)	49 (28.99)

Race	
Caucasian (%)	121 (71.6)
African American (%)	29 (17.16)
Others/unknown (%)	19 (11.24)

Coronary artery disease (%)	40 (23.67)
Hypertension (%)	96 (56.81)
Diabetes mellitus (%)	41 (24.26)
Chronic kidney disease (%)	7 (4.14)
History of tobacco (%)	85 (50.3)
History of alcohol (%)	47 (27.81)

**Table 2:** Baseline demographic characteristics for Campus 1 compared with Campus 2.

	Campus 1 (*n* = 58)	Campus 2 (*n* = 111)	*p*
	Mean (*n*)	Mean (*n*)	
Age at STEMI (95% CI)	62.75 (59.13, 66.38)	64.28 (62.12, 66.44)	0.48
BMI (95% CI)	29.62 (28.14, 31.10)	28.34 (27.41, 29.28)	0.15

Gender			
Male (%)	39 (67.24)	81 (72.97)	0.63
Female (%)	19 (32.76)	30 (27.03)	0.46

Race			
Caucasian (%)	20 (34.48)	105 (94.59)	<0.001
African American (%)	36 (62.07)	1 (0.90)	<0.001
Others/unknown (%)	2 (3.45)	5 (4.50)	0.71

Coronary artery disease (%)	12 (20.69)	28 (25.23)	0.50
Hypertension (%)	35 (60.34)	61 (54.95)	0.49
Diabetes mellitus (%)	17 (29.31)	24 (21.62)	0.28
Chronic kidney disease (%)	3 (5.17)	4 (3.60)	0.60
History of tobacco (%)	33 (56.9)	52 (46.85)	0.32
History of alcohol (%)	20 (34.83)	27 (24.32)	0.08

Calculated *p* values compare the percentage difference between campuses.

**Table 3:** Overall population metrics for door to balloon times.

	Mean	95% CI	Median	Minimum	Maximum
Door to Balloon	1:21:15	(1:18:05, 1:24:25)	1:19:00	0:43:00	2:52:00
Door to ECG	0:07:50	(0:06:29, 0:09:11)	0:06:00	0:01:00	1:09:00
ECG to page	0:09:51	(0:07:55, 0:11:47)	0:06:00	0:01:00	2:07:00
Page to table	0:29:25	(0:27:42, 0:31:08)	0:31:00	0:08:00	1:09:00
Table to Balloon	0:34:09	(0:32:19, 0:35:59)	0:32:00	0:14:00	1:40:00

Times are reported as (hours:minutes:seconds).

**Table 4:** Campus 1 (*n* = 58) and Campus 2 (*n* = 111) metrics for door-to-balloon times.

	Campus 1		Campus 2		*p* value
	Mean	95% CI	Mean	95% CI	
Door to Balloon	1:18:41	(1:13:05, 1:24:18)	1:22:46	(1:18:55, 1:26:38)	0.24
Door to ECG	0:08:11	(0:06:20, 0:10:02)	0:07:39	(0:05:49, 0:09:29)	0.68
ECG to page	0:08:32	(0:06:29, 0:10:36)	0:10:32	(0:07:48, 0:13:16)	0.25
Page to table	0:29:46	(0:26:43, 0:32:48)	0:29:26	(0:27:19, 0:31:33)	0.86
Table to balloon	0:32:12	(0:28:54, 0:35:31)	0:35:10	(0:32:59, 0:37:20)	0.15

Times are reported as hours:minutes:seconds.

**Table 5:** Minimum and maximum times for Campus 1 and Campus 2.

	Campus 1		Campus 2	
	Minimum	Maximum	Minimum	Maximum
Door to Balloon	0:44	2:20	0:43	2:52
Door to ECG	0:01	0:31	0:01	1:09
ECG to page	0:01	0:35	0:01	2:07
Page to table	0:09	1:07	0:08	1:09
Table to Balloon	0:17	1:30	0:14	1:40

Times are reported as hours:minutes.

## Data Availability

Data can be made available upon request.

## References

[1] Alpert J. S., Thygesen K., Antman E., Bassand J. P. (2000). Myocardial infarction redefined--a consensus document of the Joint European Society of Cardiology/American College of Cardiology Committee for the redefinition of myocardial infarction. *Journal of the American College of Cardiology*.

[2] Mozaffarian D., Benjamin E. J., Go A. S. (2016). Heart disease and stroke statistics—2016 update. *Circulation*.

[3] McManus D. D., Gore J., Yarzebski J., Spencer F., Lessard D., Goldberg R. J. (2011). Recent trends in the incidence, treatment, and outcomes of patients with STEMI and NSTEMI. *The American Journal of Medicine*.

[4] O’Gara P. T., Kushner F. G., Ascheim D. D. (2013). 2013 ACCF/AHA guideline for the management of ST-elevation myocardial infarction: a report of the American College of Cardiology foundation/American Heart Association task force on practice guidelines. *Circulation*.

[5] Antman E. M., Anbe D. T., Armstrong P. W. (2004). ACC/AHA guidelines for the management of patients with ST-elevation myocardial infarction--executive summary: a report of the American college of cardiology/American heart association task force on practice guidelines (writing committee to revise the 1999 guidelines for the management of patients with acute myocardial infarction). *Circulation*.

[6] O’Gara P. T., Kushner F. G., Ascheim D. D. (2013). 2013 ACCF/AHA guideline for the management of ST-elevation myocardial infarction: executive summary. *Journal of the American College of Cardiology*.

[7] Anbe D. T., Kushner F. G., Amstrong P. W. (2004). ACC/AHA guidelines for the management of patients with ST-elevation myocardial infarction. *Circulation*.

[8] De Luca G., Suryapranata H., Zijlstra F. (2003). Symptom onset-to-balloon time and mortality in patients with acute myocardial infarction treated by primary angioplasty. *Journal of the American College of Cardiology*.

[9] McNamara R. L., Wang Y., Herrin J. (2006). Effect of door-to-balloon time on mortality in patients with ST-segment elevation myocardial infarction. *Journal of the American College of Cardiology*.

[10] Brodie B. R., Gersh B. J., Stuckey T. (2010). When is door-to balloon time critical?. *Journal of the American College of Cardiology*.

[11] Brodie B. R., Hansen C., Stuckey T. D. (2006). Door-to-Balloon time with primary percutaneous coronary intervention for acute myocardial infarction impacts late cardiac mortality in high-risk patients and patients presenting early after the onset of symptoms. *Journal of the American College of Cardiology*.

[12] Brodie B. R., Stuckey T. D., Wall T. C. (1998). Importance of time to reperfusion for 30-day and late survival and recovery of left ventricular function after primary angioplasty for acute myocardial infarction. *Journal of the American College of Cardiology*.

[13] Bradley E. H., Nallamothu B. K., Herrin J. (2009). National efforts to improve door-to-balloon time. *Journal of the American College of Cardiology*.

[14] Krumholz H. M., Herrin J., Miller L. E. (2011). Improvements in door-to-balloon time in the United States, 2005 to 2010. *Circulation*.

[15] United States Census Bureau (2020). https://www.census.gov/quickfacts/fact/table/novicitymichigan,southfieldcitymichigan/PST045219.

[16] Bradley E. H., Nallamothu B. K., Curtis J. P. (2007 Sep). Summary of evidence regarding hospital strategies to reduce door-to balloon times for patients with ST-segment elevation myocardial infarction undergoing primary percutaneous coronary intervention. *Critical Pathways in Cardiology: A Journal of Evidence-Based Medicine*.

[17] Ogden C. L., Carroll M. D., Kit B. K., Flegal K. M. (2014). Prevalence of childhood and adult obesity in the United States, 2011-2012. *JAMA*.

[18] Petrelli N. J., Winer E. P., Brahmer J. (2009). Clinical cancer advances 2009: major research advances in cancer treatment, prevention, and screening—a report from the American society of clinical oncology. *Journal of Clinical Oncology*.

[19] Thornton R. L. J., Glover C. M., Cené C. W., Glik D. C., Henderson J. A., Williams D. R. (2016). Evaluating strategies for reducing health disparities by addressing the social determinants of health. *Health Affairs*.

[20] Severino P., Mather P. J., Pucci M. (2019). Advanced heart failure and end-stage heart failure: does a difference exist. *Diagnostics*.

[21] Rokos I. C., French W. J., Koenig W. J. (2009). Integration of pre-hospital electrocardiograms and ST-elevation myocardial infarction receiving center (SRC) networks. *JACC: Cardiovascular Interventions*.

[22] Peterson E. D., Dai D., DeLong E. R. (2010). Contemporary mortality risk prediction for percutaneous coronary intervention. *Journal of the American College of Cardiology*.

